# Developing criteria for evaluation of geroprotectors as a key stage toward translation to the clinic

**DOI:** 10.1111/acel.12463

**Published:** 2016-03-11

**Authors:** Alexey Moskalev, Elizaveta Chernyagina, Vasily Tsvetkov, Alexander Fedintsev, Mikhail Shaposhnikov, Vyacheslav Krut'ko, Alex Zhavoronkov, Brian K. Kennedy

**Affiliations:** ^1^ Engelhardt Institute of Molecular Biology of Russian Academy of SciencesMoscow119991Russia; ^2^ Institute of Biology of Komi Science Center of Ural Branch of Russian Academy of SciencesSyktyvkar167982Russia; ^3^ Moscow Institute of Physics and TechnologyDolgoprudny141700Russia; ^4^ The Research Institute for Translational MedicinePirogov Russian National Research Medical UniversityMoscow117997Russia; ^5^ Institute for Systems AnalysisRussian Academy of SciencesMoscow117312Russia; ^6^ D. Rogachev FRC Center for Pediatric Hematology, Oncology and ImmunologySamory Machela 1Moscow117997Russia; ^7^ The Biogerontology Research Foundation2354 Chynoweth House, Trevissome Park, Blackwater, TruroCornwallTR4 8UNUK; ^8^ Buck Institute for Research on AgingNovatoCA94945USA

**Keywords:** aging, criteria of geroprotectors, geroprotectors, healthspan, lifespan

## Abstract

In the coming decades, a massive shift in the aging segment of the population will have major social and economic consequences around the world. One way to offset this increase is to expedite the development of geroprotectors, substances that slow aging, repair age‐associated damage and extend healthy lifespan, or healthspan. While over 200 geroprotectors are now reported in model organisms and some are in human use for specific disease indications, the path toward determining whether they affect aging in humans remains obscure. Translation to the clinic is hampered by multiple issues including absence of a common set of criteria to define, select, and classify these substances, given the complexity of the aging process and their enormous diversity in mechanism of action. Translational research efforts would benefit from the formation of a scientific consensus on the following: the definition of ‘geroprotector’, the selection criteria for geroprotectors, a comprehensive classification system, and an analytical model. Here, we review current approaches to selection and put forth our own suggested selection criteria. Standardizing selection of geroprotectors will streamline discovery and analysis of new candidates, saving time and cost involved in translation to clinic.

## Introduction

Aging is a major risk factor for a number of chronic diseases, including cancer, type II diabetes, atherosclerosis, hypertension, myocardial infarction, stroke, and neurodegenerative diseases. In animal models, treatments that extend lifespan often protect against these chronic diseases and there is a reason to believe that a similar approach might work in humans. Therefore, the geroscience concept, which aims to prolong the healthy state of the human body, is likely to become a key paradigm of biomedicine in developed countries in coming decades (Seals *et al*., 2015) (http://www.nature.com/nm/journal/v21/n12/full/nm.4004.html). The aforementioned diseases and conditions may 1 day be prevented or at least delayed with the growing range of chemicals capable of slowing the processes of aging. These have been termed geroprotectors.

The search for new geroprotectors is a dynamic area of biomedicine. In their 2009 review, Kapoor *et al*. (2009) listed 24 geroprotectors known at that time. Today, more than 200 substances belong to this group, each reported to slow aging and/or increase lifespan in a variety of organisms, including yeasts, nematodes, fruit flies, and rodents, according to the Geroprotectors.org database (http://geroprotectors.org/) (Moskalev *et al*., 2015).

Despite such an impressive rate of discovery, not a single geroprotector has yet reached the pharmaceutical market as a recognized intervention targeting aging (http://www.nature.com/nm/journal/v21/n12/full/nm.4005.html). There are several nonexclusive reasons (http://www.nature.com/nm/journal/v21/n12/full/nm.4005.html). First, there is no unified mechanistic concept of aging, and primary triggers of aging are still poorly understood. As a result, molecular targets for candidate drugs are often unknown, complicating studies in the clinic. Second, there is no comprehensive system of objective human aging biomarkers. Biomarkers are critical to the translation of geroprotectors from simple model organisms to preclinical stage and then to clinic. Third, aging is not recognized as a disease or a complex of syndromes; thus, pharmaceutical companies are disinclined to create and evaluate geroprotectors (http://journal.frontiersin.org/article/10.3389/fgene.2015.00205/full, http://journal.frontiersin.org/article/10.3389/fgene.2015.00326/full). Finally, and perhaps most importantly, the scientific community has no consensus view on the concept of geroprotectors, on selection criteria for potential geroprotectors, or on the development of appropriate classification schemes, efficiency ratings, and approaches for predicting and modeling geroprotective properties. Development of such criteria would aid in the search for new geroprotectors and translate these substances into clinic more effectively.

Evidence indicates that a subset of the key causes and mechanisms of aging are ancient and evolutionarily conserved (Smith *et al*., 2007; Moskalev, 2010). Thus, targets for interventions can be identified through bioinformatics approaches and lifespan comparing data from multiple species, especially with regard to known longevity pathways. According to the GenAge database (Tacutu *et al*., 2013), there are 1825 genes whose knockout, knockdown, or overexpression is known to result in an increase in lifespan. A purposeful search of substances that affect the activity of these genes and their encoded proteins will significantly expand the pool of potential geroprotectors.

Creating a system of criteria for ranking and grouping geroprotectors in accordance with their effect on life expectancy (mean, median, maximum), their molecular targets, the mechanisms of aging involved and affected age‐associated pathologies, the transcriptomic and metabolomic changes, and the closeness of chemical structures will significantly enhance efforts to target healthspan and lifespan. Such a system will also facilitate chemical structure prediction and targeted synthesis of new geroprotectors. If this strategy can be employed successfully, it will create an alternate and cheaper route to small molecule identification compared with high‐throughput screening, which is difficult in the context of aging.

Once identified, classified, and tested, geroprotectors have great potential for the prevention and treatment of age‐related pathologies, acting on a major and common cause of these diseases – the aging process. Ultimately, they may also help in achieving a substantial extension of human healthspan, the fully active and disease‐free period of life.

In a recent review, Longo *et al*. (2015) selected a subset of the most promising interventions that could be tested in humans for their effects to slow aging and increase healthy lifespan. However, there is not complete consensus about the general principle of criteria for geroprotectors selection. In the following review, we will propose and give a detailed description of a set of primary and secondary criteria for potential geroprotectors. Providing consistency and uniformity in these areas will greatly accelerate progress in the aging research field. Based on the primary criteria, we selected potential candidates for human aging‐suppressive interventions.

## Systematic evaluation criteria for geroprotector identification

To date, more than 200 substances that extend the life of model organisms have been reported in the literature. It is evident that future studies in this direction will be more intensive, and the number of such substances will increase markedly. The emerging challenge, in turn, will be to streamline translational research to keep pace with laboratory advances. Reducing the cost and improving the efficiency with which increasingly large amounts of data from model organisms can be applied to humans will be critical to progress in the development of human geroprotectors.

At present, there are many different definitions of geroprotectors in the scientific literature. This lack of consensus creates uncertainty, especially given that significant differences exist in research methods among aging researchers, and contributes to difficulties in evaluating and comparing results. In addition, differences in experimental conditions, model organisms, and genetic background within a species further add to the difficulty of interpreting and/or comparing data. All of these issues must be weighted and taken into account in any large‐scale analysis. In this regard, we have identified the following important tasks at the present time: introduction of the concept of geroprotector and development of criteria for classifying a substance as a potential geroprotector, development of a single analytical model of a geroprotector based on these criteria, and consolidation of various research initiatives with the help of this model.

The concepts of ‘geroprotector’ and ‘gerontology’ were introduced by Ilya Mechnikov (Metchnikoff, 1910). The term ‘geroprotector’ is literally translated as an agent ‘protecting against aging’. In essence, this means ‘deterring the aging process and thus prolonging life’. Numerous synonyms including ‘anti‐aging drugs’, ‘longevity therapeutics’, ‘gerosuppressant’, ‘aging‐suppressant’, and others are also used in the scientific literature (Spindler *et al*., 2012).

For many years, the ability to increase lifespan has served as the main criterion for a geroprotector. Therefore, any substance, method, or exposure that increases lifespan is considered geroprotective. This paper focuses on substances with geroprotective properties. In real‐life situations, a researcher often has to deal with substances for which potential effects on increasing lifespan are apparent, but direct experimental evidence is lacking. For example, a substance might be known to have a positive impact on the specific mechanisms of aging or the risk of diseases associated with aging. In such cases, we propose to use the terms ‘potential geroprotector’ or ‘candidate geroprotector’. For instance, recently, a new class of drugs, senolytics, are reported to selectively kill senescent cells. Dasatinib showed notable senolytic potential, but its effects on lifespan are not yet known (Zhu *et al*., 2015).

The criteria of a geroprotector should be represented by a system that describes their desired properties and characteristics in the most complete manner. Creating such a system is not a trivial task, as it cannot be limited to one or two criteria. For example, an increase in lifespan may be accompanied by deterioration of quality of life and functional capabilities of organism, as has been reported to be the case of certain long‐lived mutants of *C. elegans* (Bansal *et al*., 2015). The real goal for translation into humans should be an increase in healthy lifespan or healthspan. Therefore, it is important to incorporate functional measures of aging wherever possible.

Thus, the task of creating a system of geroprotector criteria is becoming all the more urgent. The compliance of a substance with at least the majority of such criteria would allow the claim that a candidate drug is indeed a geroprotector. Our proposed system of criteria for geroprotectors is divided into primary and secondary groups. While the basic criteria must be met unconditionally for any candidate geroprotectors, the additional criteria can accelerate the procedures for identifying the geroprotective properties, reduce the cost of such procedures, or establish the translatability of the results into humans.

### Primary selection criteria for potential geroprotectors

#### Increased lifespan

The criterion of increasing lifespan is undoubtedly the most significant main criterion for geroprotectors. At the population level, increased lifespan manifests as reduced mortality. In an ideal situation, positive changes in all characteristics of the survival curve are observed. Those characteristics include mean lifespan, median lifespan, maximum lifespan, age of 90% mortality, and rate of aging. Many authors consider mortality rate doubling time (MRDT) as a measure of the rate of aging. This variable is derived from the Gompertz equation, MRDT = 0.693/G, where G is the exponential (Gompertz) mortality rate coefficient (Finch, 1990). An increase in MRDT is expected to reflect a decrease in the rate of aging.

In reality, the increase in lifespan is not always accompanied by positive changes in the quality of life, and a more subtle analysis of the effects of a geroprotector is required. For this reason, the introduction of additional criteria for geroprotectors is warranted and discussed below.

#### Amelioration of human aging biomarkers

Biomarkers of aging are molecular, cellular, and physiological parameters of the body that demonstrate reproducible quantitative or qualitative changes with age. Candidate geroprotectors should ideally reverse these biomarkers to a younger state or slow down the progression by which they change with age. The criterion for geroprotectors associated with biomarkers of aging is of particular importance for the translation of results into humans (Longo *et al*., 2015). Studies of human longevity under the influence of a candidate geroprotector are extremely lengthy and costly. Thus, the analysis of lifespan must be carried out on animals, but one way that the effects of a candidate geroprotector on human aging can be analyzed is on the basis of changes in various biomarkers during the geroprotective therapy.

A human aging biomarker should be minimally invasive, reproducible and should reflect main aging mechanisms. The most comprehensive list of human aging biomarkers is available online in the database Digital Ageing Atlas (http://ageing-map.org) (Craig *et al*., 2015). While there are no single definitive biomarkers for aging, a range of different measures have been proposed and are worthy of consideration. For example, when selecting geroprotectors relevant to criteria of amelioration of aging biomarkers, we can take into account the results of studies on cultures of human cells *in vitro* (expression of telomere‐related genes, beta‐amyloid‐lowering effect, low levels of advanced glycation end products and oxidative damage, reduced level of lipofuscin) or in human clinical trials (prevent neurodegeneration, hypertension, reduce blood glucose concentrations, anti‐inflammatory properties, triglyceride‐lowering effect, improve insulin sensitivity, prevent hair loss, improve immune function in the elderly, delay skin aging). Interestingly, a recent study evaluated a collection of candidate biomarkers longitudinally in a relatively young population and developed a criterion for predicting biological age that was predictive of functional parameters in 38‐year‐olds (Belsky *et al*., 2015). If these measures or a similar set prove reliable across multiple human cohorts, the testing of geroprotectors may be greatly accelerated.

#### Acceptable toxicity

Most geroprotectors show a preventive effect only when used at relatively high concentrations over long periods of time. To evaluate toxicity, studies on model organisms are commonly used (FDA 1996). Acute toxicity, characterized by median lethal dose, LD50, refers to adverse effects occurring after administration of a single dose of a substance or multiple doses given within 24 h (United Nations 2009). Other toxicity measures include identifying doses that are toxic to a specific target organ, induce carcinogenicity, reduce fertility, or increase the level of germ cell mutagenicity (United Nations 2009). Acceptable toxicity for geroprotectors should require significant (several orders of magnitude) differences between lifespan‐extending dose and toxic dose.

#### Minimal side effects at therapeutic dosage

Some substances that prolong the life of model animals in certain concentrations have multiple adverse side effects. For instance, dyslipidemia, anemia, insulin resistance, increased susceptibility to infections, hypertension, and gastrointestinal disorders have been reported in some cases. The achievement of a geroprotective effect implies in the simplest scenario the use of geroprotectors for many years. It is reasonable to expect that over the years of use some unwanted adverse effects of these drugs will be observed in addition to the expected results. These adverse effects, with passage of time, may reduce both the quality of life and the effectiveness of a geroprotector in the prevention of aging. Therefore, it is desirable to ensure that the number and severity of side effects caused by candidate geroprotectors in doses sufficient to achieve positive effects in humans is minimal.

#### Improving health‐related quality of life

The aging process is associated with decreased metabolic efficiency, as well as reduced mental and physical activity. Furthermore, an age‐related rising incidence of illnesses and disability associated with chronic diseases may significantly diminish health‐related quality of life. Potential geroprotectors should improve at least a subset of these parameters. Potential geroprotectors should improve physical, mental, emotional, and social functioning of the treated individual. Some geroprotectors are reported to stimulate cognitive function and show an antidepressant‐like effect when used in therapy, for instance preventing sleep disorders.

### Secondary selection criteria for potential geroprotector

#### Evolutionary conservatism of target or mechanism of action

An evolutionarily conserved target increases the possibility that geroprotective effects identified in simple models will reproduce in mammals. The targets of many known geroprotectors are evolutionarily conserved. For example, TOR kinase, the target of inhibition by rapamycin, is highly conserved across the range of species from unicellular yeast to humans (Dann & Thomas, 2006). Similar evolutionary conservation is evident for AMPK (Nayak *et al*., 2006), as well as NFκB (Chuang *et al*., 2013) and IGF‐1R/Akt (Song *et al*., 2014).

#### Reproducibility of geroprotective effects on different model organisms

This criterion is not identical to the previous one. Once the geroprotective effect of a substance is identified by screening for lifespan in one species, an attempt can be made to reproduce this effect in other species, even in the absence of a known conserved target. Initial screens for potential lifespan‐extending compounds are usually done in short‐lived invertebrates. Enhanced lifespan in a second invertebrate likely increases the chances that effects will also be evident in humans. Of course, data in mammals or human cells are even more valuable. In short, geroprotectors that impact lifespan in multiple animal models of aging should be given increased emphasis.

#### Simultaneous influence on several aging‐associated causes of death in mammals

Aging is driven by an intrinsic process or set of processes that enable the onset of chronic diseases. Candidate geroprotectors should be able to delay the development of one or several age‐associated pathologies. Thus, even today's geroprotective substances, which extend the lifespan in animal models, can be evaluated by their reported positive therapeutic effects on the causes of human mortality and considered for potential use as geroprotectors in humans.

#### Increase in stress resistance

At present, a large body of accumulated evidence suggests that interventions associated with enhanced longevity also often confer stress resistance (Miller, 2009). Pathways and mechanisms linked to both stress resistance and longevity include the insulin/IGF‐1 (Longo & Fabrizio, 2002) (Holzenberger *et al*., 2003), TOR (Lin *et al*., 2014) and NF‐κB (Helenius *et al*., 1996) signaling pathways, DNA repair (Moskalev *et al*., 2013), free radicals detoxication (Cutler, 2005), molecular chaperones (Morley & Morimoto, 2004), and epigenetic control of gene expression (Saunders & Verdin, 2009). In their recent review, Epel and Lithgow (Epel & Lithgow, 2014) suggested that reduction in stress resistance is a common feature for all of the nine hallmarks of aging proposed earlier by López‐Otín *et al*. (2013). These hallmarks include the loss of proteostasis, epigenetic changes, genomic instability, telomere attrition, altered intercellular communication, dysregulated nutrient sensing, mitochondrial dysfunction, cell senescence, and stem cell exhaustion. Hence, it is a reasonable assumption that potential geroprotectors act to increase the body's resistance to adverse environmental factors. As with other categories, we do not rule out the possibility that a geroprotector will not confer stress resistance; however, we believe this to be a strong enough correlation to date to include in our analytics. Thus, the criterion of increasing stress resistance can serve as one of indicators of the drug's specific activating effect on the mechanisms of longevity associated with the resistance to stress.

## Identification of candidates

Longevity studies on animal models and human aging studies can provide sufficient support to initiate clinical trials. Limited investigations have been performed to date, however. The impact of the mTOR inhibitor RAD001 (Everolimus) on immunosenescence was evaluated, and it has shown promising effects (Mannick *et al*., 2014). Another study proved metformin was able to reduce diabetes‐related and all‐cause mortality, myocardial infarction, and any diabetes‐related end point in individuals with type 2 diabetes (Scarpello, 2003; Wang *et al*., 2014). Recently scientists have set a new clinical trial to investigate whether metformin would be able to reduce mortality of healthy adults (https://clinicaltrials.gov/show/NCT02432287).

According to our analysis of the literature, approximately 200 compounds that can extend the lifespan of animal models have now been discovered. The extent to which they comply with the proposed criteria described above varies widely. Analysis of published data with the use of developed criteria did, however, reveal candidates that fit all of the main criteria (e.g., acarbose, deprenyl, d‐glucosamine, dihydroergocristine methanesulfonate, ellagic acid, fenofibrate, glutathione, metformin, spermidine, tyrosol, and vinpocetine), and we suggest that they are tractable candidates for human interventions. Notably, we have compiled data in the literature for this analysis and have tried to impose minimal judgments on data quality. While this has the advantage of reducing bias imposed by the authors, readers are encouraged to examine the direct literature for specific compounds of interest.

### Acarbose

Acarbose is an α‐glucosidase inhibitor used to treat diabetes mellitus. Treated with this compound, male UM‐HET3 mice had 22% longer lifespan (Harrison *et al*., 2014). It has acceptable acute toxicity – mouse LD50 oral is 24 gm kg^−1^ (Tomasulo, 2002). Acarbose is considered to be well‐tolerable drug with adverse effects not significantly differing from placebo (Hotta *et al*., 1993). Acarbose reduces blood glucose concentrations, blood pressure, triglycerides, the progression of intima media thickness, and incidence of cardiovascular events and of newly diagnosed hypertension, and it demonstrates beneficial effects on overweight individuals and downregulates biomarkers of low‐grade inflammation (Hanefeld & Schaper, 2008).

### Deprenyl

Deprenyl is a selective MAO‐B inhibitor used to treat major depression and early‐stage Parkinson disease. It prolongs lifespan in male rat (Kitani *et al*., 1993). No significant acute toxicological data for this compound were identified in literature search (mouse LD50 intraperitoneal 200 mg kg^−1^) (Kane *et al*., 1988). Deprenyl is also well tolerated (Robottom, 2011). Deprenyl protects human neurons from apoptosis induced by various kinds of insults by interfering with early apoptotic signaling events and may be applicable to delay the deterioration of neurons during advancing aging (Maruyama & Naoi, 1999; Magyar *et al*., 2004).

### D‐glucosamine

D‐glucosamine is an amino sugar and a potent drug for the treatment of arthritis, although its effectiveness is controversial (Burdett & McNeil, 2012). Nevertheless, it is able to promote lifespan of nematodes and C57BL/6NRj mice (Weimer *et al*., 2014). This compound has very low acute toxicity and is safe for everyday use (Reginster *et al*., 2001). Current use of glucosamine was associated with a significant decreased risk of death from cancer (HR 0.87 95% CI 0.76‐0.98) and with a large risk reduction for death from respiratory diseases (HR 0.59 95% CI 0.41‐0.83) (Pocobelli *et al*., 2010; Bell *et al*., 2012). Glucosamine supplementation can significantly decrease risk of lung cancer in humans (Brasky *et al*., 2011). A meta‐analysis has shown that glucosamine has lowest risk of adverse effects compared with other treatments (Diarecin and NSAIDs) (Kongtharvonskul *et al*., 2015). The oral supplementation of glucosamine can potentially improve cutaneous aging in human and reduce the appearance of visible wrinkles and fine lines of the skin (Murad & Tabibian, 2001).

### Dihydroergocristine methanesulfonate

Dihydroergocristine methanesulfonate is an fungally derived alkaloid salt and a potent vasodilator (Valli *et al*., 1984). It is one of the 60 compounds identified in a screen for increased longevity in *C. elegans* (Ye *et al*., 2014). In two studies (Aranda *et al*., 1992; Milvio, 1992), dihydroergocristine was shown as safe and well tolerated with rare side effects including mild gastralgia, nausea, and dyspepsia in aged patients with senile dementia of Alzheimer type. Dihydroergocristine methanesulfonate in some studies has statistically significant positive effects on symptoms of age‐related cognitive dysfunction (Wadworth & Chrisp, 1992).

### Ellagic acid

Ellagic acid is a natural polyphenol found in numerous edible plants. Its ability to prolong *C. elegans* lifespan is proposed to be due to a hormetic effect (Saul *et al*., 2011). This substance has also low acute toxicity – rat LD50 oral > 20 gm kg^−1^ (Tomasulo, 2002). Study on rats showed a potentially good tolerability (Tasaki *et al*., 2008). Ellagic acid prevents collagen destruction and inflammatory responses caused by UV‐B‐induced photoaging in HaCaT keratinocytes and human dermal fibroblasts (Bae *et al*., 2010).

### Glutathione

Glutathione is a tripeptide with notable antioxidant effect. Glutathione prevents aging‐associated oxidation of proteins in the crystalline lens (Kamei, 1993).It is also able to promote *C. elegans* lifespan (Shibamura *et al*., 2009) and has rather low acute toxicity – mouse LD50 oral 5 gm kg^−1^ (Tomasulo, 2002). A recent study showed that glutathione was well tolerated with few side effects (Richie *et al*., 2015).

### Metformin

Metformin is an oral antidiabetic drug of the biguanide class that is widely prescribed as an early‐stage drug for type II diabetes. It enhances longevity is a range of model organisms: *C. elegans* (Cabreiro *et al*., 2013), *D. melanogaster* (Slack *et al*., 2012), and *M. musculus* (Martin‐Montalvo *et al*., 2013). However, it should be noted that there are studies showing no effect or only a very small effect of metformin in rats and rodents with normal genetics and longevity (https://www.ncbi.nlm.nih.gov/pubmed/20304770, http://www.ncbi.nlm.nih.gov/pubmed/23900241). It has acceptable acute toxicity – mouse LD50 oral 1450 mg kg^−1^ (Tomasulo, 2002) – and is well tolerated (Giugliano *et al*., 1993). Serious but rare adverse effects include lactic acidosis (mostly linked to alcoholism due to depletion of NAD+ stores), heart failure, respiratory disease (due to inadequate oxygenation of tissues), and impaired renal function. Metformin treatment confers insulin sensitivity, leads to weight loss, and improves lipid profiles (Salpeter *et al*., 2008).

### Rapamycin

Rapamycin is an antifungal agent with immunosuppressive and antiproliferative effects. It has a lifespan‐prolonging effect in several studies on different model organisms, including *S. cerevisiae* (Powers *et al*., 2006; Medvedik *et al*., 2007), *C. elegans* (Robida‐Stubbs *et al*., 2012), *D. melanogaster* (Moskalev & Shaposhnikov, 2010), and *M. musculus* (Harrison *et al*., 2009; Fok *et al*., 2014; Miller *et al*., 2014). It has acceptable acute toxicity – mouse LD50 oral > 2500 mg kg^−1^ (Vezina *et al*., 1975) – although it can induce a range of reversible but concerning side effects (Soefje *et al*., 2011). Other rapamycin derivatives (termed rapalogs) appear to have similar effects. A majority of reports also indicate that rapamycin delays pathologies of aging in mice and protects against age‐related diseases, including cardiac and neurodegenerative syndromes, as well as neoplasms (Johnson *et al*., 2013; Lamming *et al*., 2013). Rapamycin also is reported to suppress nuclear defects in cells isolated from Hutchinson–Gilford progeria syndrome, although reports of the drug have not been reported in mouse models of the disease (Oyanagui, 1984; Pocobelli *et al*., 2010).^67,68^

### Spermidine

Spermidine is a natural polyamine compound found in animal tissues. It is reported to promote lifespan of yeast, flies, worms, human cells, and mice (Eisenberg *et al*., 2009). Spermidine has acceptable acute toxicity – mouse LD30 oral 1 gm kg^−1^ (Oyanagui, 1984). Administration of spermidine, whose intracellular concentration declines during human aging, markedly extends the lifespan of human immune cells. In aging human cells, spermidine treatment triggers epigenetic deacetylation of histone H3 through inhibition of histone acetyltransferases, enhances autophagic flux, and suppresses oxidative stress and necrosis (Eisenberg *et al*., 2009).

### Tyrosol

Tyrosol is natural phenolic antioxidant, a derivative of phenethil alcohol. It is able to increase both mean and maximum lifespan in *C. elegans* (Canuelo *et al*., 2012) and is well tolerated (Tuck & Hayball, 2002). Tyrosol can afford considerable protection against aging‐associated heart deterioration (Owen *et al*., 2000). Several studies showed a cardioprotective role of tyrosol (Samuel *et al*., 2008; Smol'iakova *et al*., 2010; Sun *et al*., 2015). Tyrosol's cardiovascular benefits are likely due to its ability to prevent oxidation of LDL (Covas *et al*., 2006; Castaner *et al*., 2012). Also, tyrosol ameliorated hyperglycemia by regulating key enzymes of carbohydrate metabolism in streptozotocin‐induced diabetic rats (Chandramohan *et al*., 2015). A human study of tyrosol‐rich products (white wine and virgin olive oil) demonstrated an anti‐inflammatory effect (Migliori *et al*., 2015). Tyrosol has a potent anti‐allergic effect by inhibiting the degranulation of mast cells and expression of inflammatory cytokines (Je *et al*., 2015). Tyrosol has very low acute toxicity (LD50 was found to be 2700 and 1700 mg kg^−1^ in mice after intragastric and intraperitoneal injection, respectively, and 7079 mg kg^−1^ in rats after intragastric administration). No toxicity was observed after 3 months of chronic intragastric administration of p‐tyrosol at the doses of 200 mg kg^−1^ in male rats and 10 mg kg^−1^ in dogs (Saratikov & Krasnov, 2004).

### Vinpocetine

Vinpocetine is a semisynthetic derivative of the alkaloid vincamine with nootropic effects. It is another of the 60 compounds identified in a recent screen for enhanced longevity in *C. elegans* (Ye *et al*., 2014). It has acceptable acute toxicity – mouse LD50 oral 534 mg kg^−1^ (Cholnoky & Domok, 1976). In an 18‐month study, vinpocetine was reported safe and had a good tolerability during the whole study period (Valikovics *et al*., 2012). In another study, vinpocetine failed to show effectiveness in treatment Alzheimer disease, but there also were no significant side effects from drug therapy (Thal *et al*., 1989). Vinpocetine significantly inhibits *in vitro* aging of human erythrocytes (Bayer *et al*., 1988).

Identification of potential geroprotectors will be even more effective with the development of geroprotector criteria, which involves the building of appropriate databases (Moskalev *et al*., 2015). A number of additional requirements related to the storage, data format, access control, request processing, etc., in these databases should be met. In the future, knowledge bases should have the capability to process the custom queries in natural language, reveal lists of important criteria and assessments that directly affect the lifespan of the subjects (patients), and compare the major rules obtained by different methods.

### Nonpharmacological interventions

There are also several nonpharmacological interventions affecting lifespan and aging biomarkers. Among them are physical exercise (PE) and caloric restriction (CR). CR delays aging both in rodents and primates (http://tpx.sagepub.com/content/24/6/742.full.pdf) and improves aging biomarkers in humans including BMI, systolic and diastolic blood pressure, glucose, insulin, and lipids (http://biomedgerontology.oxfordjournals.org/content/5). Moreover, CR decreases risks of aging‐associated diseases, such as diabetes and cardiovascular disease (http://www.ncbi.nlm.nih.gov/pubmed/10630589). However, this intervention has potential side effects on human health, which limits its implementation (http://www.sciencedirect.com/science/article/pii/S004). In humans, CR side effects may be lowered by intermittent fasting (http://www.ncbi.nlm.nih.gov/pmc/articles/PMC3946160/).

Intermittent fasting (IF) is a pattern of eating that cycle between a period of fasting and nonfasting. CR and IF have similar effects on cardiovascular biomarkers (http://www.fasebj.org/content/20/6/631.full). CR and IF effects may be caused by reduction in specific nutrients, for example, proteins and especially amino acids such as methionine (https://www.ncbi.nlm.nih.gov/pubmed/8429371, http://online.liebertpub.com/doi/abs/10.1089/rej.2009) or tryptophane.

As methionine restriction effects also depend on other factors (http://journals.plos.org/plosgenetics/article?id=10.1, http://www.fasebj.org/cgi/content/meeting_abstract/25, http://www.ncbi.nlm.nih.gov/pubmed/19956092/), we suppose that combination of this diet with geroprotectors may have a cumulative effect on extending lifespan.

In animal models, PE has shown ability to increase survival, but not the maximal lifespan (http://www.ncbi.nlm.nih.gov/pubmed/1542046, http://www.ncbi.nlm.nih.gov/pubmed/4055572). Nevertheless, PE has impact on healthspan by decreasing risk of age‐related diseases, including cardiovascular disease, diabetes, and osteoporosis (http://www.ncbi.nlm.nih.gov/pubmed/22210414). Moreover, it may be used in combination with CR to improve both maximal lifespan and healthspan (http://www.ncbi.nlm.nih.gov/pubmed/9049716).

## Discussion

Our goal is to establish a set of meaningful criteria by which to promote the identification of successful geroprotectors. Positive identification of candidates would help to direct efforts and resources, maximizing the chances of identifying interventions that extend human healthspan.

It should be noted that all currently known geroprotectors are characterized by relatively weak effectiveness. A positive impact on lifespan rarely exceeds 40% in invertebrates and is often less in mammals (Spindler, 2012; Lucanic *et al*., 2013). In contrast, much larger effects have been observed with genetic interventions, for instance a 10‐fold enhancement induced by mutations in a PI3K subunit gene (Ayyadevara *et al*., 2008). It is possible that the most promising path to increase the efficiency of geroprotectors lies in the increase in their specificity to molecular targets. High specificity and efficiency may be achievable through a combination of targeting and screening approaches.

Increasing abundance of genomic, transcriptomic, proteomic, metabolomic, and metagenomic data, together with the development of bioinformatics approaches, makes geroprotector screening approaches based on searching for gene signatures, identifying aging‐associated pathways more efficient. These *in silico* approaches may lead to the development of new classes and combinations of geroprotectors that would have a more robust effect on health span and lifespan. Early attempts at using metabolic transformation algorithms aiming to mimic the young metabolic state using known drugs showed promising results (Yizhak *et al*., 2013). Approaches using large‐scale human multi‐omic data derived from healthy patients of various ages and patients with pathologies and data linked to the effects of large number of drugs are currently being explored (Zhavoronkov *et al*., 2014).

At present, the view that aging is a systemic process represented by a set of several overlapping pathways is increasingly taking hold. In our opinion, it is worth discussing the pathological networks of genes of aging. Affecting the most important nodes of such networks would likely provide geroprotection. Therefore, the traditional target‐based approach may be surpassed by systems‐based approaches, with the goal of influencing a combination of several targets that are of key importance in the aging process (Carretero *et al*., 2015).

From this perspective, it may be advisable to combine known geroprotectors along with the development of new drugs. As aging involves many intracellular signaling pathways, the joint action of several geroprotectors aimed at different targets or processes may have an additive or synergistic effect on life expectancy. For example, according to our earlier data, the combined effect of TOR, PI3K, and NF‐kB pathway inhibitors increased *Drosophila* lifespan to a greater extent than the individual drugs alone (Danilov *et al*., 2013). In another example, the combined action of TOR and JNK inhibitors on the rotifer *Brachionus manjavacas* increased the average lifespan by an additional 65% compared with each inhibitor alone (Snell *et al*., 2014). Thus, use of the right combinations of geroprotectors may lead to a substantially greater pharmacological effect on life expectancy.

Another aspect important when testing geroprotectors individually and in combinations is personalization and tissue specificity. While many geroprotectors are expected to be efficacious across multiple species in heterogeneous populations, side effects may vary significantly. Ideal geroprotectors regimen should include a set of companion diagnostic markers to ensure personalization on the tissue‐specific and system levels and adjusted to multiple parameters including age, gender, and lifestyle.

There is also the possibility of repurposing older medications for new geroprotective indications (Ye *et al*., 2014). The most effective cures for old age may already be on the market, but they remain unknown because their geroprotective properties have not yet been studied. If such drugs are already approved by the FDA, it makes for an easier path to test them in humans. Metformin may be a classic example and a recent retrospective study of diabetic patients taking metformin indicated that the drug might be delaying the onset of other aging pathologies (Bannister *et al*., 2014). Such approaches could save substantial time and resources usually spent on drug testing and approval. Accepted hallmarks for geroprotectors can play a leading role in this process.

## Funding

Russian Science Foundation (Grant/Award Number: 14‐50‐00060). B.K.K. is an Ellison Medical Foundation Senior Scholar in Aging.

## Conflict of interest

The authors declare no potential conflict of interests.

## Supporting information


**Table S1** Summary of the effects on lifespan, healthspan, and potential side effects of the pharmacological geroprotector candidates, ordered on the base of evolutionary conservation of lifespan effects in different models.

## References

[acel12463-bib-0001] Aranda B , Dumoulin P , Groothold G (1992) Controlled study of the effect of dihydroergocristine on organic brain psychosyndrome. Arzneimittelforschung 42, 1406–1409. 1492863

[acel12463-bib-0002] Ayyadevara S , Alla R , Thaden JJ , Shmookler Reis RJ (2008) Remarkable longevity and stress resistance of nematode PI3K‐null mutants. Aging Cell 7, 13–22. 17996009 10.1111/j.1474-9726.2007.00348.x

[acel12463-bib-0003] Bae JY , Choi JS , Kang SW , Lee YJ , Park J , Kang YH (2010) Dietary compound ellagic acid alleviates skin wrinkle and inflammation induced by UV‐B irradiation. Exp. Dermatol. 19, e182–e190. 20113347 10.1111/j.1600-0625.2009.01044.x

[acel12463-bib-0004] Bannister CA , Holden SE , Jenkins‐Jones S , Morgan CL , Halcox JP , Schernthaner G , Mukherjee J , Currie CJ (2014) Can people with type 2 diabetes live longer than those without? A comparison of mortality in people initiated with metformin or sulphonylurea monotherapy and matched, non‐diabetic controls. Diabetes Obes. Metab. 16, 1165–1173. 25041462 10.1111/dom.12354

[acel12463-bib-0005] Bansal A , Zhu LJ , Yen K , Tissenbaum HA (2015) Uncoupling lifespan and healthspan in *Caenorhabditis elegans* longevity mutants. Proc. Natl Acad. Sci. USA 112, E277–E286. 25561524 10.1073/pnas.1412192112 PMC4311797

[acel12463-bib-0006] Bayer R , Plewa S , Borcescu E , Claus W (1988) Filterability of human erythrocytes – drug induced prevention of aging *in vitro* . Arzneimittelforschung 38, 1765–1767. 3245847

[acel12463-bib-0007] Bell GA , Kantor ED , Lampe JW , Shen DD , White E (2012) Use of glucosamine and chondroitin in relation to mortality. Eur. J. Epidemiol. 27, 593–603. 22828954 10.1007/s10654-012-9714-6 PMC3557824

[acel12463-bib-0008] Belsky DW , Caspi A , Houts R , Cohen HJ , Corcoran DL , Danese A , Harrington H , Israel S , Levine ME , Schaefer JD , Sugden K , Williams B , Yashin AI , Poulton R , Moffitt TE (2015) Quantification of biological aging in young adults. Proc. Natl Acad. Sci. USA 112, E4104–E4110. 26150497 10.1073/pnas.1506264112 PMC4522793

[acel12463-bib-0009] Brasky TM , Lampe JW , Slatore CG , White E (2011) Use of glucosamine and chondroitin and lung cancer risk in the VITamins And Lifestyle (VITAL) cohort. Cancer Causes Control 22, 1333–1342. 21706174 10.1007/s10552-011-9806-8 PMC3175750

[acel12463-bib-0010] Burdett N , McNeil JD (2012) Difficulties with assessing the benefit of glucosamine sulphate as a treatment for osteoarthritis. Int. J. Evid. Based Healthc. 10, 222–226. 22925619 10.1111/j.1744-1609.2012.00279.x

[acel12463-bib-0011] Cabreiro F , Au C , Leung KY , Vergara‐Irigaray N , Cocheme HM , Noori T , Weinkove D , Schuster E , Greene ND , Gems D (2013) Metformin retards aging in *C. elegans* by altering microbial folate and methionine metabolism. Cell 153, 228–239. 23540700 10.1016/j.cell.2013.02.035 PMC3898468

[acel12463-bib-0012] Canuelo A , Gilbert‐Lopez B , Pacheco‐Linan P , Martinez‐Lara E , Siles E , Miranda‐Vizuete A (2012) Tyrosol, a main phenol present in extra virgin olive oil, increases lifespan and stress resistance in *Caenorhabditis elegans* . Mech. Ageing Dev. 133, 563–574. 22824366 10.1016/j.mad.2012.07.004

[acel12463-bib-0013] Carretero M , Gomez‐Amaro RL , Petrascheck M (2015) Pharmacological classes that extend lifespan of *Caenorhabditis elegans* . Front. Genet. 6, 77. 25784926 10.3389/fgene.2015.00077 PMC4347486

[acel12463-bib-0014] Castaner O , Covas MI , Khymenets O , Nyyssonen K , Konstantinidou V , Zunft HF , de la Torre R , Munoz‐Aguayo D , Vila J , Fito M (2012) Protection of LDL from oxidation by olive oil polyphenols is associated with a downregulation of CD40‐ligand expression and its downstream products *in vivo* in humans. Am. J. Clin. Nutr. 95, 1238–1244. 22440854 10.3945/ajcn.111.029207

[acel12463-bib-0015] Chandramohan R , Pari L , Rathinam A , Sheikh BA (2015) Tyrosol, a phenolic compound, ameliorates hyperglycemia by regulating key enzymes of carbohydrate metabolism in streptozotocin induced diabetic rats. Chem. Biol. Interact. 229, 44–54. 25641191 10.1016/j.cbi.2015.01.026

[acel12463-bib-0016] Cholnoky E , Domok LI (1976) Summary of safety tests of ethyl apovincaminate. Arzneimittelforschung 26, 1938–1944. 1037220

[acel12463-bib-0017] Chuang KH , Peng YC , Chien HY , Lu ML , Du HI , Wu YL (2013) Attenuation of LPS‐induced lung inflammation by glucosamine in rats. Am. J. Respir. Cell Mol. Biol. 49, 1110–1119. 23898954 10.1165/rcmb.2013-0022OC

[acel12463-bib-0018] Covas MI , de la Torre K , Farre‐Albaladejo M , Kaikkonen J , Fito M , Lopez‐Sabater C , Pujadas‐Bastardes MA , Joglar J , Weinbrenner T , Lamuela‐Raventos RM , de la Torre R (2006) Postprandial LDL phenolic content and LDL oxidation are modulated by olive oil phenolic compounds in humans. Free Radic. Biol. Med. 40, 608–616. 16458191 10.1016/j.freeradbiomed.2005.09.027

[acel12463-bib-0019] Craig T , Smelick C , Tacutu R , Wuttke D , Wood SH , Stanley H , Janssens G , Savitskaya E , Moskalev A , Arking R , de Magalhaes JP (2015) The Digital Ageing Atlas: integrating the diversity of age‐related changes into a unified resource. Nucleic Acids Res. 43, D873–D878. 25232097 10.1093/nar/gku843 PMC4384002

[acel12463-bib-0020] Cutler RG (2005) Oxidative stress and aging: catalase is a longevity determinant enzyme. Rejuvenation Res. 8, 138–140. 16144468 10.1089/rej.2005.8.138

[acel12463-bib-0021] Danilov A , Shaposhnikov M , Plyusnina E , Kogan V , Fedichev P , Moskalev A (2013) Selective anticancer agents suppress aging in *Drosophila* . Oncotarget 4, 1527–1546. 24096697 10.18632/oncotarget.1272 PMC3824538

[acel12463-bib-0022] Dann SG , Thomas G (2006) The amino acid sensitive TOR pathway from yeast to mammals. FEBS Lett. 580, 2821–2829. 16684541 10.1016/j.febslet.2006.04.068

[acel12463-bib-0023] Eisenberg T , Knauer H , Schauer A , Buttner S , Ruckenstuhl C , Carmona‐Gutierrez D , Ring J , Schroeder S , Magnes C , Antonacci L , Fussi H , Deszcz L , Hartl R , Schraml E , Criollo A , Megalou E , Weiskopf D , Laun P , Heeren G , Breitenbach M , Grubeck‐Loebenstein B , Herker E , Fahrenkrog B , Frohlich K‐U , Sinner F , Tavernarakis N , Minois N , Kroemer G , Madeo F (2009) Induction of autophagy by spermidine promotes longevity. Nat. Cell Biol. 11, 1305–1314. 19801973 10.1038/ncb1975

[acel12463-bib-0024] Epel ES , Lithgow GJ (2014) Stress biology and aging mechanisms: toward understanding the deep connection between adaptation to stress and longevity. J. Gerontol. A Biol. Sci. Med. Sci. 69(Suppl 1), S10–S16. 24833580 10.1093/gerona/glu055 PMC4022128

[acel12463-bib-0025] FDA (1996) Guidance for Industry: Single Dose Acute Toxicity Testing for Pharmaceuticals. Silver Spring: Center for Drug Evaluation and Research (CDER).

[acel12463-bib-0026] Finch CE (1990) Longevity, Senescence, and the Genome. Chicago and London: University of Chicago Press.

[acel12463-bib-0027] Fok WC , Chen Y , Bokov A , Zhang Y , Salmon AB , Diaz V , Javors M , Wood WH 3rd , Zhang Y , Becker KG , Perez VI , Richardson A (2014) Mice fed rapamycin have an increase in lifespan associated with major changes in the liver transcriptome. PLoS ONE 9, e83988. 24409289 10.1371/journal.pone.0083988 PMC3883653

[acel12463-bib-0028] Giugliano D , Quatraro A , Consoli G , Minei A , Ceriello A , De Rosa N , D'Onofrio F (1993) Metformin for obese, insulin‐treated diabetic patients: improvement in glycaemic control and reduction of metabolic risk factors. Eur. J. Clin. Pharmacol. 44, 107–112. 8453955 10.1007/BF00315466

[acel12463-bib-0029] Hanefeld M , Schaper F (2008) Acarbose: oral anti‐diabetes drug with additional cardiovascular benefits. Expert Rev. Cardiovasc. Ther. 6, 153–163. 18248270 10.1586/14779072.6.2.153

[acel12463-bib-0030] Harrison DE , Strong R , Sharp ZD , Nelson JF , Astle CM , Flurkey K , Nadon NL , Wilkinson JE , Frenkel K , Carter CS , Pahor M , Javors MA , Fernandez E , Miller RA (2009) Rapamycin fed late in life extends lifespan in genetically heterogeneous mice. Nature 460, 392–395. 19587680 10.1038/nature08221 PMC2786175

[acel12463-bib-0031] Harrison DE , Strong R , Allison DB , Ames BN , Astle CM , Atamna H , Fernandez E , Flurkey K , Javors MA , Nadon NL , Nelson JF , Pletcher S , Simpkins JW , Smith D , Wilkinson JE , Miller RA (2014) Acarbose, 17‐alpha‐estradiol, and nordihydroguaiaretic acid extend mouse lifespan preferentially in males. Aging Cell 13, 273–282. 24245565 10.1111/acel.12170 PMC3954939

[acel12463-bib-0032] Helenius M , Hanninen M , Lehtinen SK , Salminen A (1996) Aging‐induced up‐regulation of nuclear binding activities of oxidative stress responsive NF‐kB transcription factor in mouse cardiac muscle. J. Mol. Cell. Cardiol. 28, 487–498. 9011632 10.1006/jmcc.1996.0045

[acel12463-bib-0033] Holzenberger M , Dupont J , Ducos B , Leneuve P , Geloen A , Even PC , Cervera P , Le Bouc Y (2003) IGF‐1 receptor regulates lifespan and resistance to oxidative stress in mice. Nature 421, 182–187. 12483226 10.1038/nature01298

[acel12463-bib-0034] Hotta N , Kakuta H , Sano T , Matsumae H , Yamada H , Kitazawa S , Sakamoto N (1993) Long‐term effect of acarbose on glycaemic control in non‐insulin‐dependent diabetes mellitus: a placebo‐controlled double‐blind study. Diabet. Med. 10, 134–138. 8458189 10.1111/j.1464-5491.1993.tb00030.x

[acel12463-bib-0035] Je IG , Kim DS , Kim SW , Lee S , Lee HS , Park EK , Khang D , Kim SH (2015) Tyrosol suppresses allergic inflammation by inhibiting the activation of phosphoinositide 3‐kinase in mast cells. PLoS ONE 10, e0129829. 26068872 10.1371/journal.pone.0129829 PMC4465982

[acel12463-bib-0036] Johnson SC , Rabinovitch PS , Kaeberlein M (2013) mTOR is a key modulator of ageing and age‐related disease. Nature 493, 338–345. 23325216 10.1038/nature11861 PMC3687363

[acel12463-bib-0037] Kamei A (1993) Glutathione levels of the human crystalline lens in aging and its antioxidant effect against the oxidation of lens proteins. Biol. Pharm. Bull. 16, 870–875. 8268853 10.1248/bpb.16.870

[acel12463-bib-0038] Kane JM , Dudley MW , Sorensen SM , Miller FP (1988) 2,4‐Dihydro‐3H‐1,2,4‐triazole‐3‐thiones as potential antidepressant agents. J. Med. Chem. 31, 1253–1258. 3373495 10.1021/jm00401a031

[acel12463-bib-0039] Kapoor VK , Dureja J , Chadha R (2009) Synthetic drugs with anti‐ageing effects. Drug Discov. Today 14, 899–904. 19638318 10.1016/j.drudis.2009.07.006

[acel12463-bib-0040] Kitani K , Kanai S , Sato Y , Ohta M , Ivy GO , Carrillo MC (1993) Chronic treatment of (‐)deprenyl prolongs the life span of male Fischer 344 rats. Further evidence. Life Sci. 52, 281–288. 8423709 10.1016/0024-3205(93)90219-s

[acel12463-bib-0041] Kongtharvonskul J , Anothaisintawee T , McEvoy M , Attia J , Woratanarat P , Thakkinstian A (2015) Efficacy and safety of glucosamine, diacerein, and NSAIDs in osteoarthritis knee: a systematic review and network meta‐analysis. Eur. J. Med. Res. 20, 24. 25889669 10.1186/s40001-015-0115-7 PMC4359794

[acel12463-bib-0042] Lamming DW , Ye L , Sabatini DM , Baur JA (2013) Rapalogs and mTOR inhibitors as anti‐aging therapeutics. J. Clin. Invest. 123, 980–989. 23454761 10.1172/JCI64099 PMC3582126

[acel12463-bib-0043] Lin YH , Chen YC , Kao TY , Lin YC , Hsu TE , Wu YC , Ja WW , Brummel TJ , Kapahi P , Yuh CH , Yu LK , Lin ZH , You RJ , Jhong YT , Wang HD (2014) Diacylglycerol lipase regulates lifespan and oxidative stress response by inversely modulating TOR signaling in *Drosophila* and *C. elegans* . Aging Cell 13, 755–764. 24889782 10.1111/acel.12232 PMC4116436

[acel12463-bib-0044] Longo VD , Fabrizio P (2002) Regulation of longevity and stress resistance: a molecular strategy conserved from yeast to humans? Cell. Mol. Life Sci. 59, 903–908. 12169020 10.1007/s00018-002-8477-8 PMC11337509

[acel12463-bib-0045] Longo VD , Antebi A , Bartke A , Barzilai N , Brown‐Borg HM , Caruso C , Curiel TJ , de Cabo R , Franceschi C , Gems D , Ingram DK , Johnson TE , Kennedy BK , Kenyon C , Klein S , Kopchick JJ , Lepperdinger G , Madeo F , Mirisola MG , Mitchell JR , Passarino G , Rudolph KL , Sedivy JM , Shadel GS , Sinclair DA , Spindler SR , Suh Y , Vijg J , Vinciguerra M , Fontana L (2015) Interventions to slow aging in humans: are we ready? Aging Cell 14, 497–510. 25902704 10.1111/acel.12338 PMC4531065

[acel12463-bib-0046] López‐Otín C , Blasco MA , Partridge L , Serrano M , Kroemer G (2013) The hallmarks of aging. Cell 153, 1194–1217. 23746838 10.1016/j.cell.2013.05.039 PMC3836174

[acel12463-bib-0047] Lucanic M , Lithgow GJ , Alavez S (2013) Pharmacological lifespan extension of invertebrates. Ageing Res. Rev. 12, 445–458. 22771382 10.1016/j.arr.2012.06.006 PMC3552093

[acel12463-bib-0048] Magyar K , Palfi M , Tabi T , Kalasz H , Szende B , Szoko E (2004) Pharmacological aspects of (‐)‐deprenyl. Curr. Med. Chem. 11, 2017–2031. 15279565 10.2174/0929867043364793

[acel12463-bib-0049] Mannick JB , Del Giudice G , Lattanzi M , Valiante NM , Praestgaard J , Huang B , Lonetto MA , Maecker HT , Kovarik J , Carson S , Glass DJ , Klickstein LB (2014) mTOR inhibition improves immune function in the elderly. Sci. Transl. Med. 6, 268ra179. 10.1126/scitranslmed.3009892 25540326

[acel12463-bib-0050] Martin‐Montalvo A , Mercken EM , Mitchell SJ , Palacios HH , Mote PL , Scheibye‐Knudsen M , Gomes AP , Ward TM , Minor RK , Blouin MJ , Schwab M , Pollak M , Zhang Y , Yu Y , Becker KG , Bohr VA , Ingram DK , Sinclair DA , Wolf NS , Spindler SR , Bernier M , de Cabo R (2013) Metformin improves healthspan and lifespan in mice. Nat. Commun. 4, 2192. 23900241 10.1038/ncomms3192 PMC3736576

[acel12463-bib-0051] Maruyama W , Naoi M (1999) Neuroprotection by (‐)‐deprenyl and related compounds. Mech. Ageing Dev. 111, 189–200. 10656536 10.1016/s0047-6374(99)00066-4

[acel12463-bib-0052] Medvedik O , Lamming DW , Kim KD , Sinclair DA (2007) MSN2 and MSN4 link calorie restriction and TOR to sirtuin‐mediated lifespan extension in *Saccharomyces cerevisiae* . PLoS Biol. 5, e261. 17914901 10.1371/journal.pbio.0050261 PMC1994990

[acel12463-bib-0053] Metchnikoff E , Mitchell PC (1910) The Prolongation of Life: Optimistic Studies. New and Revised Edition. New York & London: G. P. Putnam's Sons.

[acel12463-bib-0054] Migliori M , Panichi V , de la Torre R , Fito M , Covas M , Bertelli A , Munoz‐Aguayo D , Scatena A , Paoletti S , Ronco C (2015) Anti‐inflammatory effect of white wine in CKD patients and healthy volunteers. Blood Purif. 39, 218–223. 25833063 10.1159/000371570

[acel12463-bib-0055] Miller RA (2009) Cell stress and aging: new emphasis on multiplex resistance mechanisms. J. Gerontol. A Biol. Sci. Med. Sci. 64, 179–182. 19225033 10.1093/gerona/gln072 PMC2655020

[acel12463-bib-0056] Miller RA , Harrison DE , Astle CM , Fernandez E , Flurkey K , Han M , Javors MA , Li X , Nadon NL , Nelson JF , Pletcher S , Salmon AB , Sharp ZD , Van Roekel S , Winkleman L , Strong R (2014) Rapamycin‐mediated lifespan increase in mice is dose and sex dependent and metabolically distinct from dietary restriction. Aging Cell 13, 468–477. 24341993 10.1111/acel.12194 PMC4032600

[acel12463-bib-0057] Milvio C (1992) Dihydroergocristine in the treatment of organic brain psychosyndrome. Dose‐finding study against placebo. Arzneimittelforschung 42, 1399–1402. 1492861

[acel12463-bib-0058] Morley JF , Morimoto RI (2004) Regulation of longevity in *Caenorhabditis elegans* by heat shock factor and molecular chaperones. Mol. Biol. Cell 15, 657–664. 14668486 10.1091/mbc.E03-07-0532 PMC329286

[acel12463-bib-0059] Moskalev AA (2010) Evolution conceptions about the nature of aging. Adv. Gerontol. 23, 9–20. 20586244

[acel12463-bib-0060] Moskalev AA , Shaposhnikov MV (2010) Pharmacological inhibition of phosphoinositide 3 and TOR kinases improves survival of *Drosophila melanogaster* . Rejuvenation Res. 13, 246–247. 20017609 10.1089/rej.2009.0903

[acel12463-bib-0061] Moskalev AA , Shaposhnikov MV , Plyusnina EN , Zhavoronkov A , Budovsky A , Yanai H , Fraifeld VE (2013) The role of DNA damage and repair in aging through the prism of Koch‐like criteria. Ageing Res. Rev. 12, 661–684. 22353384 10.1016/j.arr.2012.02.001

[acel12463-bib-0062] Moskalev A , Chernyagina E , de Magalhães JP , Barardo D , Thoppil H , Shaposhnikov M , Budovsky A , Fraifeld VE , Garazha A , Tsvetkov V , Bronovitsky E , Bogomolov V , Scerbacov A , Kuryan O , Gurinovich R , Jellen LC , Kennedy B , Mamoshina P , Dobrovolskaya E , Aliper A , Kaminsky D , Zhavoronkov A (2015) Geroprotectors.org: a new, structured and curated database of current therapeutic interventions in aging and age‐related disease. Aging (Albany NY) 7, 616–628. 26342919 10.18632/aging.100799 PMC4600621

[acel12463-bib-0063] Murad H , Tabibian MP (2001) The effect of an oral supplement containing glucosamine, amino acids, minerals, and antioxidants on cutaneous aging: a preliminary study. J. Dermatolog. Treat. 12, 47–51. 12171689 10.1080/095466301750163590

[acel12463-bib-0064] Nayak V , Zhao K , Wyce A , Schwartz MF , Lo WS , Berger SL , Marmorstein R (2006) Structure and dimerization of the kinase domain from yeast Snf1, a member of the Snf1/AMPK protein family. Structure 14, 477–485. 16531232 10.1016/j.str.2005.12.008

[acel12463-bib-0065] Owen RW , Giacosa A , Hull WE , Haubner R , Wurtele G , Spiegelhalder B , Bartsch H (2000) Olive‐oil consumption and health: the possible role of antioxidants. Lancet Oncol. 1, 107–112. 11905662 10.1016/s1470-2045(00)00015-2

[acel12463-bib-0066] Oyanagui Y (1984) Anti‐inflammatory effects of polyamines in serotonin and carrageenan paw edemata – possible mechanism to increase vascular permeability inhibitory protein level which is regulated by glucocorticoids and superoxide radical. Agents Actions 14, 228–237. 6324559 10.1007/BF01966647

[acel12463-bib-0067] Pocobelli G , Kristal AR , Patterson RE , Potter JD , Lampe JW , Kolar A , Evans I , White E (2010) Total mortality risk in relation to use of less‐common dietary supplements. Am. J. Clin. Nutr. 91, 1791–1800. 20410091 10.3945/ajcn.2009.28639 PMC2869514

[acel12463-bib-0068] Powers RW 3rd , Kaeberlein M , Caldwell SD , Kennedy BK , Fields S (2006) Extension of chronological life span in yeast by decreased TOR pathway signaling. Genes Dev. 20, 174–184. 16418483 10.1101/gad.1381406 PMC1356109

[acel12463-bib-0069] Reginster JY , Deroisy R , Rovati LC , Lee RL , Lejeune E , Bruyere O , Giacovelli G , Henrotin Y , Dacre JE , Gossett C (2001) Long‐term effects of glucosamine sulphate on osteoarthritis progression: a randomised, placebo‐controlled clinical trial. Lancet 357, 251–256. 11214126 10.1016/S0140-6736(00)03610-2

[acel12463-bib-0070] Richie JP Jr , Nichenametla S , Neidig W , Calcagnotto A , Haley JS , Schell TD , Muscat JE (2015) Randomized controlled trial of oral glutathione supplementation on body stores of glutathione. Eur. J. Nutr. 54, 251–263. 24791752 10.1007/s00394-014-0706-z

[acel12463-bib-0071] Robida‐Stubbs S , Glover‐Cutter K , Lamming DW , Mizunuma M , Narasimhan SD , Neumann‐Haefelin E , Sabatini DM , Blackwell TK (2012) TOR signaling and rapamycin influence longevity by regulating SKN‐1/Nrf and DAF‐16/FoxO. Cell Metab. 15, 713–724. 22560223 10.1016/j.cmet.2012.04.007 PMC3348514

[acel12463-bib-0072] Robottom BJ (2011) Efficacy, safety, and patient preference of monoamine oxidase B inhibitors in the treatment of Parkinson's disease. Patient Prefer. Adherence 5, 57–64. 21423589 10.2147/PPA.S11182 PMC3058602

[acel12463-bib-0073] Salpeter SR , Buckley NS , Kahn JA , Salpeter EE (2008) Meta‐analysis: metformin treatment in persons at risk for diabetes mellitus. Am. J. Med. 121, 149–157.e2. 18261504 10.1016/j.amjmed.2007.09.016

[acel12463-bib-0074] Samuel SM , Thirunavukkarasu M , Penumathsa SV , Paul D , Maulik N (2008) Akt/FOXO3a/SIRT1‐mediated cardioprotection by n‐tyrosol against ischemic stress in rat *in vivo* model of myocardial infarction: switching gears toward survival and longevity. J. Agric. Food Chem. 56, 9692–9698. 18826227 10.1021/jf802050h PMC2648870

[acel12463-bib-0075] Saratikov AS , Krasnov EA (2004) Rhodiola Rosea (Golden Root): A Valuable Medicinal Plant. Tomsk: Tomsk University Press.

[acel12463-bib-0076] Saul N , Pietsch K , Sturzenbaum SR , Menzel R , Steinberg CE (2011) Diversity of polyphenol action in *Caenorhabditis elegans*: between toxicity and longevity. J. Nat. Prod. 74, 1713–1720. 21805983 10.1021/np200011a

[acel12463-bib-0077] Saunders LR , Verdin E (2009) Cell biology. Stress response and aging. Science 323, 1021–1022. 19229027 10.1126/science.1170007

[acel12463-bib-0078] Scarpello JHB (2003) Improving survival with metformin: the evidence base today. Diabetes Metab. 29, 6S36–6S43. 14502099 10.1016/s1262-3636(03)72786-4

[acel12463-bib-0079] Seals DR , Justice JN , LaRocca TJ (2015) Physiological geroscience: targeting function to increase healthspan and achieve optimal longevity. J. Physiol. doi: 10.1113/jphysiol.2014.282665 10.1113/jphysiol.2014.282665 PMC4933122 25639909

[acel12463-bib-0080] Shibamura A , Ikeda T , Nishikawa Y (2009) A method for oral administration of hydrophilic substances to *Caenorhabditis elegans*: effects of oral supplementation with antioxidants on the nematode lifespan. Mech. Ageing Dev. 130, 652–655. 19580823 10.1016/j.mad.2009.06.008

[acel12463-bib-0081] Slack C , Foley A , Partridge L (2012) Activation of AMPK by the putative dietary restriction mimetic metformin is insufficient to extend lifespan in *Drosophila* . PLoS ONE 7, e47699. 23077661 10.1371/journal.pone.0047699 PMC3473082

[acel12463-bib-0082] Smith ED , Kennedy BK , Kaeberlein M (2007) Genome‐wide identification of conserved longevity genes in yeast and worms. Mech. Ageing Dev. 128, 106–111. 17126379 10.1016/j.mad.2006.11.017

[acel12463-bib-0083] Smol'iakova VI , Chernyshova GA , Plotnikov MB , Aliev OI , Krasnov EA (2010) [Antioxidant and cardioprotective effects of N‐tyrosol in myocardial ischemia with reperfusion in rats]. Kardiologiia 50, 47–49. 21526564

[acel12463-bib-0084] Snell TW , Johnston RK , Rabeneck B , Zipperer C , Teat S (2014) Joint inhibition of TOR and JNK pathways interacts to extend the lifespan of *Brachionus manjavacas* (Rotifera). Exp. Gerontol. 52, 55–69. 24486130 10.1016/j.exger.2014.01.022 PMC3970784

[acel12463-bib-0085] Soefje SA , Karnad A , Brenner AJ (2011) Common toxicities of mammalian target of rapamycin inhibitors. Target. Oncol. 6, 125–129. 21499766 10.1007/s11523-011-0174-9

[acel12463-bib-0086] Song KH , Kang JH , Woo JK , Nam JS , Min HY , Lee HY , Kim SY , Oh SH (2014) The novel IGF‐IR/Akt‐dependent anticancer activities of glucosamine. BMC Cancer 14, 31. 24438088 10.1186/1471-2407-14-31 PMC3901559

[acel12463-bib-0087] Spindler SR (2012) Review of the literature and suggestions for the design of rodent survival studies for the identification of compounds that increase health and life span. Age (Dordr) 34, 111–120. 21424790 10.1007/s11357-011-9224-6 PMC3260350

[acel12463-bib-0088] Spindler SR , Li R , Dhahbi JM , Yamakawa A , Sauer F (2012) Novel protein kinase signaling systems regulating lifespan identified by small molecule library screening using *Drosophila* . PLoS ONE 7, e29782. 22363408 10.1371/journal.pone.0029782 PMC3282711

[acel12463-bib-0089] Sun L , Fan H , Yang L , Shi L , Liu Y (2015) Tyrosol prevents ischemia/reperfusion‐induced cardiac injury in H9c2 cells: involvement of ROS, Hsp70, JNK and ERK, and apoptosis. Molecules 20, 3758–3775. 25723850 10.3390/molecules20033758 PMC6272375

[acel12463-bib-0090] Tacutu R , Craig T , Budovsky A , Wuttke D , Lehmann G , Taranukha D , Costa J , Fraifeld VE , de Magalhaes JP (2013) Human Ageing Genomic Resources: integrated databases and tools for the biology and genetics of ageing. Nucleic Acids Res. 41, D1027–D1033. 23193293 10.1093/nar/gks1155 PMC3531213

[acel12463-bib-0091] Tasaki M , Umemura T , Maeda M , Ishii Y , Okamura T , Inoue T , Kuroiwa Y , Hirose M , Nishikawa A (2008) Safety assessment of ellagic acid, a food additive, in a subchronic toxicity study using F344 rats. Food Chem. Toxicol. 46, 1119–1124. 18155344 10.1016/j.fct.2007.10.043

[acel12463-bib-0092] Thal LJ , Salmon DP , Lasker B , Bower D , Klauber MR (1989) The safety and lack of efficacy of vinpocetine in Alzheimer's disease. J. Am. Geriatr. Soc. 37, 515–520. 2715559 10.1111/j.1532-5415.1989.tb05682.x

[acel12463-bib-0093] Tomasulo P (2002) ChemIDplus – super source for chemical and drug information. Med. Ref. Serv. Q. 21, 53–59. 11989279 10.1300/J115v21n01_04

[acel12463-bib-0094] Tuck KL , Hayball PJ (2002) Major phenolic compounds in olive oil: metabolism and health effects. J. Nutr. Biochem. 13, 636–644. 12550060 10.1016/s0955-2863(02)00229-2

[acel12463-bib-0095] United Nations (2009) Health hazards In Globally Harmonized System of Classification and Labelling of Chemicals (GHS)). New York and Geneva: United Nations, pp 107–212.

[acel12463-bib-0096] Valikovics A , Csanyi A , Nemeth L (2012) Study of the effects of vinpocetin on cognitive functions. Ideggyogy Sz. 65, 115–120. 23136730

[acel12463-bib-0097] Valli P , Zucca G , Seghezzi R , Botta L (1984) Effect of various vasoactive drugs on synaptic transmission in the orthosympathetic ganglia in man. Boll. Soc. Ital. Biol. Sper. 60, 1191–1197. 6089853

[acel12463-bib-0098] Vezina C , Kudelski A , Sehgal SN (1975) Rapamycin (AY‐22,989), a new antifungal antibiotic. I. Taxonomy of the producing streptomycete and isolation of the active principle. J. Antibiot. 28, 721–726. 1102508 10.7164/antibiotics.28.721

[acel12463-bib-0099] Wadworth AN , Chrisp P (1992) Co‐dergocrine mesylate. A review of its pharmacodynamic and pharmacokinetic properties and therapeutic use in age‐related cognitive decline. Drugs Aging 2, 153–173. 1606351 10.2165/00002512-199202030-00002

[acel12463-bib-0100] Wang CP , Lorenzo C , Espinoza SE (2014) Frailty attenuates the impact of metformin on reducing mortality in older adults with type 2 diabetes. J. Endocrinol. Diabetes Obes. 2, 1031. 25506599 PMC4264048

[acel12463-bib-0101] Weimer S , Priebs J , Kuhlow D , Groth M , Priebe S , Mansfeld J , Merry TL , Dubuis S , Laube B , Pfeiffer AF , Schulz TJ , Guthke R , Platzer M , Zamboni N , Zarse K , Ristow M (2014) D‐Glucosamine supplementation extends life span of nematodes and of ageing mice. Nat. Commun. 5, 3563. 24714520 10.1038/ncomms4563 PMC3988823

[acel12463-bib-0102] Ye X , Linton JM , Schork NJ , Buck LB , Petrascheck M (2014) A pharmacological network for lifespan extension in *Caenorhabditis elegans* . Aging Cell 13, 206–215. 24134630 10.1111/acel.12163 PMC3955372

[acel12463-bib-0103] Yizhak K , Gabay O , Cohen H , Ruppin E (2013) Model‐based identification of drug targets that revert disrupted metabolism and its application to ageing. Nat. Commun. 4, 2632. 24153335 10.1038/ncomms3632

[acel12463-bib-0104] Zhavoronkov A , Buzdin AA , Garazha AV , Borisov NM , Moskalev AA (2014) Signaling pathway cloud regulation for *in silico* screening and ranking of the potential geroprotective drugs. Front. Genet. 5, 49. 24624136 10.3389/fgene.2014.00049 PMC3940060

[acel12463-bib-0105] Zhu Y , Tchkonia T , Pirtskhalava T , Gower AC , Ding H , Giorgadze N , Palmer AK , Ikeno Y , Hubbard GB , Lenburg M , O'Hara SP , LaRusso NF , Miller JD , Roos CM , Verzosa GC , LeBrasseur NK , Wren JD , Farr JN , Khosla S , Stout MB , McGowan SJ , Fuhrmann‐Stroissnigg H , Gurkar AU , Zhao J , Colangelo D , Dorronsoro A , Ling YY , Barghouthy AS , Navarro DC , Sano T , Robbins PD , Niedernhofer LJ , Kirkland JL (2015) The Achilles' heel of senescent cells: from transcriptome to senolytic drugs. Aging Cell 14, 644–658. 25754370 10.1111/acel.12344 PMC4531078

