# Supplementary material for: Developing criteria for evaluation of geroprotectors as a key stage toward translation to the clinic
Source: Aging Cell. 2016 Mar 11;15(3):407–15. doi: 10.1111/acel.12463 (PMC4854916; doi:10.1111/acel.12463)
Supplement: Supplementary file 1 — Table S1 Summary of the effects on lifespan, healthspan, and potential side effects of the pharmacological geroprotector candidates, ordered on the base of evolutionary conservation of lifespan effects in different models. [file ACEL-15-407-s001.pdf]

Table 1 Summary of the effects on lifespan, healthspan, and potential side effects of the pharmacological geroprotector candidates, ordered on the base of evolutionary conservation of lifespan effects in different models

Geroprotector Candidate	Primary selection criteria																Affects aging-associated diseases				
	Reproducibility of anti-aging effect in different models				Amelioration of human aging biomarkers							Low toxicity	Low risk side effects	Quality of life. Improvement of functioning							
	Yeast	Worm	Flies	Rodents	Anti Amiloid	Anti Inflammation	Anti hyperglycemia	Antioxidation	Improves Lipid Profile	Neuroprotection	Improve Cutaneous Aging			Physical	Mental	Emotional	Anti Cancer	Anti Atherogenic	Anti Diabetic	Anti Cardiovascular	Alzheimer Disease
**Acarbose**				+		+	+		+			+	+	+					+	+	
**Deprenyl**				+					+			+	+			+	+				
**D-glucosamine**		+		+		+				+		+	+	+			+	+			
**Dihydroergocristine methanesulfonate**		+								+		+	+		+						
**Ellagic acid**		+			+	+				+	+	+	+			+	+	+	+	+	
**Glutathione**		+						+				+	+								
**Metformin**		+	0	+		+	+		+	+		+	+	+	+	+	+	+	+	+	
**Rapamycin**	+		+	+	+	+				+		+	–		+		+	+		+	
**Spermidine**	+	+	+					+				+	+							+	
**Tyrosol**		+					+					+	+	+						+	
**Vinpocetine**		+								+		+	+		+						+

+ - positive effect; – - negative effect; 0 - no effect; empty cell - no data available

References:

**Acarbose**	(Hotta *et al.* 1993; Hanefeld & Schaper 2008; Nakhaee & Sanjari 2013; Harrison *et al.* 2014; Holman *et al.* 2014; Wang *et al.* 2014; McCarty & DiNicolantonio 2015)
**Deprenyl**	(Kitani *et al.* 1993; ThyagaRajan *et al.* 2000; Tatton *et al.* 2003)
**D-glucosamine**	(Hua *et al.* 2002; Park *et al.* 2009; Kaida *et al.* 2014; Weimer *et al.* 2014; Kongtharvonskul *et al.* 2015)
**Dihydroergocristine methanesulfonate**	(Ye *et al.* 2014)
**Ellagic acid**	(Feng *et al.* 2009; Saul *et al.* 2011; Dhingra & Chhillar 2012; Muthenna *et al.* 2012; Uzar *et al.* 2012; Kannan & Quine 2013; Panchal *et al.* 2013; Ding *et al.* 2014; El-Garhy *et al.* 2014; Zhang *et al.* 2014)
**Glutathione**	(Shibamura *et al.* 2009; Veljkovic *et al.* 2012)
**Metformin**	(Slack *et al.* 2012; Cabreiro *et al.* 2013; Martin-Montalvo *et al.* 2013; Eriksson & Nystrom 2014; Gong *et al.* 2014; Guo *et al.* 2014; Lee *et al.* 2014; Ohira *et al.* 2014; Patil *et al.* 2014; Ashabi *et al.* 2015)
**Rapamycin**	(Alvers *et al.* 2009; Gadioli *et al.* 2009; Moskalev & Shaposhnikov 2010; Robida-Stubbs *et al.* 2012; Cai & Yan 2013; Flynn *et al.* 2013; Ashworth & Wu 2014; Ehninger *et al.* 2014; Fok *et al.* 2014; Xu *et al.* 2014; Song *et al.* 2015)
**Spermidine**	(Eisenberg *et al.* 2009; LaRocca *et al.* 2013)
**Tyrosol**	(Owen *et al.* 2000; Canuelo *et al.* 2012; Wang *et al.* 2013; Chandramohan *et al.* 2015)
**Vinpocetine**	(Herrera-Mundo & Sitges 2013; Ye *et al.* 2014)
